# The gravity of an edge

**DOI:** 10.1007/s41109-018-0063-6

**Published:** 2018-05-10

**Authors:** Mary E. Helander, Sarah McAllister

**Affiliations:** 1 IBM T. J. Watson Research Center, Applied Data Science, P.O. Box 218, Yorktown Heights, 10598 NY USA; 2 CUNY Borough of Manhattan Community College, Department of Mathematics, 199 Chambers Street, New York, 10007 NY USA

**Keywords:** Bridges to nowhere, Edge betweenness, *k*-shortest path (KSP), Edge Gravity, *k*-Gravity, Node centrality, Path enumeration, Social network analysis, Strength of weak ties, Structural importance, Ties that bind

## Abstract

We describe a methodology for characterizing the relative structural importance of an arbitrary network edge by exploiting the properties of a *k*-shortest path algorithm. We introduce the metric *Edge Gravity*, measuring how often an edge occurs in any possible network path, as well as *k*-Gravity, a lower bound based on paths enumerated while solving the *k*-shortest path problem. The methodology is demonstrated using Granovetter’s original *strength of weak ties* network examples as well as the well-known Florentine families of the Italian Renaissance and the Krebs 2001 terrorist networks. The relationship to edge betweenness is established. It is shown that important edges, i.e. ones with a high *Edge Gravity*, are not necessarily adjacent to nodes of importance as identified by standard centrality metrics, and that key nodes, i.e. ones with high centrality, often have their importance bolstered by being adjacent to *bridges to nowhere*–e.g. ones with low *Edge Gravity*. It is also demonstrated that *Edge Gravity* distinguishes critically important bridges or local bridges from those of lesser structural importance.

## Introduction

Much attention has been given to assessing the importance of actors–i.e., the nodes–in a social network. This focus coincides with norms of contemporary Western culture, which place a higher value on individual achievement, competition, and personal prowess than on cooperative achievement, collaboration, and the forging of strong relationships. In this paper, we eschew node importance and instead consider the question, How important is an arbitrary network edge? Motivating our interest is the fact that relationships are the very essence of social networks; the interpretation of an edge as a relationship tie or link between *people* distinguishes social networks from the graphs used to model and analyze transportation systems, the Internet, scheduling and sequencing problems, and various other applications.

The notion of social network edge importance has existed throughout history, although it has not always been studied algorithmically via network models and analytics. Consider, for example, cultures that practice arranged marriages, where matchmaking is a means of connecting families to ensure the passage of wealth, establish power, perpetuate family business interests, and so forth (Hussain 1999). In a social setting, structural changes typically occur when an existing relationship is severed (divorce, the end of a friendship, leaving an organization) or a new one created (marriage, making a new friend, professional networking). Relationships are often easier to form or dissolve in groups where people remain fairly constant. On the other hand, adding or removing nodes–actors in the social network–is more difficult and often carries a higher cost, whether that be the hiring process in business or the loss of an individual in a personal network. Quantification of the importance of a relationship, either existing or proposed–independent of the importance of the actors–appears to be useful for the maintenance of existing relationships and the cultivation of new ones.

### The edge importance problem

This work was inspired by the research question, How important is an arbitrary individual edge in a network? After thoughtful consideration, we realized that first and foremost, meaningful quantification of edge importance, distinct and separate from node centrality, is essential.

In social network theory, *edge betweenness*–a metric based on shortest paths–is the predominant metric for characterizing the importance of a relationship between actors. Other notable notions of edge importance to network structure are those of *bridges* and *local bridges*. The designation of a bridge is a form of edge structural importance, albeit a binary one. The mathematical concept of bridging was first introduced by Harary et al. (1965). For undirected graphs, an edge (*i*,*j*) is a bridge when (*i*,*j*) is the *only* path from *i* to *j*. The removal of a bridge disconnects a network. The concept of local bridging was explored in a sociological context by Granovetter (1983, 1973). Granovetter remarked that true bridges are unlikely in large social networks, where alternate paths are common. However, such alternate paths may be rather long and thus inefficient routes for information transmission. A *local bridge* is an edge whose removal would not necessarily disconnect the network but would still be significantly disruptive to information flow. More precisely, local bridges are edges (*i*,*j*) where the next shortest path from *i* to *j* has a length of at least three. Hence, local bridges facilitate information flow between parts of the network that would otherwise be distant and difficult to reach.

Granovetter (1973) further noted that every local bridge is a “weak tie”, though not every weak tie is a local bridge. Hence, weak ties in a social network can play an important role in information diffusion when they serve as local bridges between more well-connected portions of the network. The key idea is that actors with strong ties are likely to have many mutual friends and to be privy to the same information flow, and hence do not bridge the gaps between disparate groups. On the other hand, weak ties are more likely to be effective paths for disseminating new information between groups of individuals that have strong ties to one another within, but not between, the groups. Today, the idea that weak ties are most significant in helping job seekers to successfully find employment is a well-accepted notion in social network theory. (See, for example, Jackson (2005) or Granovetter (1995)).

Granovetter (1973) remarked that local bridges earn their significance by creating more paths. This is based on the idea put forward by Davies (1966) that information flow from one person to another is directly proportional to the quantity of all possible paths between those two actors–unlike with transportation, the Internet, scheduling and sequencing, and various other network applications where paths play an important role in route planning. The insights of Granovetter and Davies are consistent with the notion that information flow in a social network is rarely prescribed along a designated path in advance–as might be the case in route planning, for example, when navigating a vehicle from a point of origin to a destination on a roadway network.

Following the insights of Granovetter and Davies, the concept of *Edge Gravity* emerged as a natural metric that can be computed algorithmically as the number of paths in a network that rely on a particular edge. Edge Gravity extends the notion of *edge betweenness* while elevating appreciation for edges that are involved in alternate paths (of any length). Indeed, Edge Gravity provides a way of assessing the structural importance of an edge based on how often that edge appears in *any* path, rather than restricting attention only to shortest paths, as is the case with edge betweenness.

Our methodology provides a systematic approach to identifying and ranking the most structurally important edges in a given network and is based on observing and quantifying the number of times a particular edge appears in any possible network path. For networks where all paths can be enumerated, *Edge Gravity* can be computed exactly in polynomial time by an adaptation of the *k*-shortest path algorithm. For networks where not all paths can be enumerated, a lower bound for *Edge Gravity* is found in polynomial time by the same algorithmic approach. In either case, the gravity of an edge, or its lower bound, is useful for ranking the importance of an edge, leading to an exact or estimated ranking respectively.

We were motivated by the remark of Granovetter that *local bridges earn their significance by creating more paths.* We tested this hypothesis to see whether Edge Gravity could effectively identify and rank the local bridges of Granovetter’s examples. Our results were positive; in fact, they indicate that it is very practical to use path enumeration to quantify and rank relative edge importance.

Due to the uncertain nature of information flow in a social network, more paths generally mean more transmission efficiency–a notion that is mathematically consistent with established research in the field of network reliability and communications. See, for example, Ball (1980) and Colbourn (1987), who also note the NP– and *♯*P–completeness of problems and computations where full-path enumeration is required for exactness. Here, we leverage Miaou and Chin’s observation (Miaou and Chin 1991) that a *k*-shortest path algorithm may find all paths for a large network. They observed that solving the *k*-shortest path problem can be used for generating alternate paths in large transportation networks and showed that sometimes (depending on the selection of *k*), a polynomial time algorithm ends up enumerating all paths in the network.

We exploit these properties of *k*-shortest path algorithms for computing the *Edge Gravity* metric exactly, or finding a lower bound (called, *k*-Gravity) to indicate the relative importance of a network edge. We infer from Eppstein’s work (Eppstein 1998) that solving the *k*-shortest path problem while enumerating all possible network paths is possible in, for example, *O*(*m*+*n* log*n*+*kn*) time. As is common in graph theoretical applications, we sidestep the computational issues posed by an underlying intractable problem (in our case, complete path enumeration) by noticing that most of the time, the worst case is rare. (See, for example, Chandrasekaran et al. (2008) and Liu et al. (2012)).

### General motivation and domain relevance

*Edge Gravity* and its implications for structural importance of social network components is relevant to several application domains where finding and prioritizing edges may help to solve entrenched societal problems. For example, proposing new edges or isolating existing ones could be a promising way to advance diplomacy or public policy when *echo chambers* may be responsible for perpetuating divisive attitudes. Conversely, finding important existing or potential edges between otherwise isolated populations is already known to be helpful in quarantining fast-spreading infectious diseases. In general, *Edge Gravity* offers a quantification and ranking approach that may be helpful for new sociological studies aimed at improving the deeper understanding of how relationships pertain to the effectiveness of human activities such as job-seeking, fund-raising, and more.

### Paper organization

The rest of the paper is organized as follows. The next section details the methodology: an algorithm for quantifying the importance (gravity) of a network edge. We also show how the ranking of edges by gravity leads to straightforward ways to identify important local bridges (i.e., a subset of Granovetter’s weak ties), which we nickname *ties that bind*, as well as structurally unimportant bridges, which we nickname *bridges to nowhere*. We apply the algorithm to a small network in order to illustrate all the mechanical steps of the methodology, including path enumeration. The third section, Case Studies, applies the Edge Gravity Algorithm and associated methodology to four well-known social networks: two of Granovetter’s examples (Granovetter 1973), the Florentine families network of the Italian Renaissance (Wasserman and Faust 1999), and the Krebs terrorist network (Krebs 2002). The fourth section revisits the case study examples from the perspective of comparing the Edge Gravity analysis to that of established edge metrics. In the final section, we compare and contrast our findings with those of other researchers, past and present, and offer a summary of the main points of this paper, general conclusions, and suggestions for future work.

## Methodology

This section describes the methodology used to quantify edges, either exactly or as a lower bound, and then rank them, either exactly or approximately, by their importance as measured by *gravity*. The gravity labels are then used to isolate an edge set that we have nicknamed *ties that bind*. We also discuss how isolating the *ties that bind* helps to distinguish bridges of lesser importance, which we have nicknamed *bridges to nowhere*. We show that the algorithm produces its results very efficiently.

### Definitions and notation

Noting that we can convert any undirected network to a digraph by replacing each undirected edge with a directed arc in each direction, we develop the more general methodology for digraphs. We begin with the following definitions: Edge Gravity is a non-negative integer value calculated for a specific edge, representing the number of times the edge occurs in any network path. For a digraph *D* (*V*,*A*) with vertex set *V* and arc set *A*, we denote *ℓ* (*a*) as the gravity label for an arc *a*∈*A*. Edge Gravity Ranking is found by ordering the arc set *A* according to the descending value of *ℓ* (*a*). Ties that Bind is the subset of edges that have a high gravity value. We call the most important edges, identified by the gravity edge metric, *ties that bind* because they are the edges that locally bridge portions of a network which might otherwise be disconnected or difficult to reach. Bridges to Nowhere is the subset of edges that are *bridges* but have a low gravity value. Bridges are typically considered important because the removal of a bridge disconnects the network. However, the disconnection caused by the removal of a bridge in this set is less important because the disconnection is only to a relatively small component (e.g., a single node or a small node subset).

Miaou and Chin (1991) showed that it is possible to use a *k*-shortest path algorithm to generate alternate paths for large road transportation networks. A side result of Miaou and Chin (1991) was the discovery that given a sufficiently large value of *k*, the complete collection of all possible network paths can often be obtained in polynomial time. For the undirected case, edge labels are formed by summing the replacement arc labels.

Let *k*^∗^ denote the smallest *k* for which implementing the *k*-shortest path algorithm may produce all possible network paths. When the *k*-shortest path algorithm terminates by finding at most the *h*-shortest paths, with *h*<*k*, for every origin-destination pair, then *k*^∗^ is observable. In fact, this termination condition indicates that all paths have been enumerated and that *k*^∗^ is equal to the value of *h*. The *Edge Gravity Algorithm* described in the next section determines all the gravity labels for edges while finding *k*^∗^ when *k* is adequately large. When *k*^∗^ is not found, the labels can be used to find lower bounds for Edge Gravity and an approximate edge ranking.

### The Edge Gravity Algorithm

Given digraph *D* (*V*,*A*) with node set *V*, arc set *A*, *n*=|*V*|, *m*=|*A*|, and positive integer *k*, the following algorithm creates a non-negative integer label *ℓ* (*a*) for each *a*∈*A* and specifies the value of *k*^∗^ if found. Initialization Step: Set *ℓ* (*a*)←0 for all *a*∈*A*, and set *k*^∗^←*Undefined*. For each *r*∈*V*, repeat the main steps: Main Steps: Step 1: Find the *k*-shortest paths from root node *r* to every other node *i*∈*V*. Step 2: For each path identified in **Step 1**, let $\mathcal {P} \subseteq A$ denote the set of arcs in the path. For each $a \in \mathcal {P}$, set *ℓ* (*a*)←*ℓ* (*a*)+1. Step 3: Let *k*_*r*_=max_*i*∈*V*_[*k*_*r*,*i*_], where *k*_*r*,*i*_ is the number of paths found from root node *r* to node *i*, for all *i*∈*V*. Final Step: Let $\tilde {k}=\text {max}_{r \in V} [ k_{r}]$. If $\tilde {k} < k$, then $k^{\ast } \longleftarrow \tilde {k}$. (In other words, all paths have been enumerated.) Otherwise, we know that *k*^∗^≥*k* must be true, but *k*^∗^ remains *Undefined*.

Because the dominant step is finding the *k*-shortest paths (i.e., Step 1), the algorithm complexity follows from the *k*-shortest path algorithm applied–for example, *O*(*m*+*n* log*n*+*kn*) following Eppstein (1998). Furthermore, note that *any* algorithm for solving the *k*-shortest path problem may be used for this step. In fact, since the earlier works in this area by Miaou and Chin (1991) and Eppstein (1998), the *k*-shortest path problem has received much attention, including more recently by Bhosle (2005), Hershberger et al. (2007), Feng (2014), Kurz and Mutzel (2016), and Wen et al. (2017).

The set of *bridges to nowhere* is easily identified by recursively removing edges which have one endpoint node with degree one. The *ties that bind*, while not as precisely defined, are the collection of edges with the largest Edge Gravity. Like edge betweenness, Edge Gravity favors edges that connect dense subgroups. The number to include in this group is similar in concept to that of the number of recalculations of edge betweenness and removal in Newman and Girvan’s (2004) approach for finding community structure in networks.

#### Translation of Edge Gravity labels to undirected edges

The algorithm can be applied to an undirected graph *G* (*V*,*E*), where *V* is the set of nodes and *E* is the set of undirected edges, in the following way. For each edge (*i*,*j*)∈*E*, we create two arcs, *a*≡(*i*,*j*) and $\grave {a} \equiv (j,i)$, in the set *A*. The output of the Edge Gravity Algorithm provides labels for arcs *ℓ* (*a*) and $\ell \; (\grave {a})$, which are the *arc gravity* metrics for each arc. The *Edge Gravity* label is the sum of the two replacement arcs *ℓ* (*a*) and $\ell \; (\grave {a})$.

#### Interpreting Edge Gravity labels when *k*^∗^ is found

Note that Miaou and Chin (1991) demonstrated that *k*-shortest path algorithms were capable of generating large sets of alternate paths, sometimes finding and enumerating all paths. Eppstein (1998) showed that the *k*-shortest path could be solved on a digraph *D* (*V*,*A*) in *O* (*m*+*n* log*n*+*kn*) time. The Edge Gravity Algorithm enumerates all paths in the digraph *D* (*V*,*A*) if it terminates with *k*^∗^ defined. In this case, the arc label *ℓ* (*a*) indicates the number of times the arc is included over all possible paths in the network.

#### Interpreting Edge Gravity labels when *k*^∗^ is not found

For the examples and case studies of this paper, we are able to find *k*^∗^ when the Edge Gravity Algorithm terminates by successively increasing the value of *k*. For dense social networks that have large numbers of paths of equal length, it may be that *k* grows arbitrarily large or that computational resources are exhausted. Since path enumeration belongs to the class of *♯*P–complete computation problems, it may be that the Edge Gravity Algorithm is unable to enumerate all paths or to terminate with a definite *k*^∗^ value. However, if *k*^∗^ remains undefined, then the gravity arc labels *ℓ* (*a*) are useful for computing valid lower bounds on the true number of times the arc is included over all possible paths in the network. This is because at least a subset of all paths is revealed.

Note that when *k*^∗^ is found, then for undirected networks, *ℓ* (*a*) and $\ell \; (\grave {a})$ measure the use of the edge by all paths, in one direction and then the other, and thus will be equal. If *k*^∗^ remains *Undefined*, then a greater lower bound on an edge’s gravity is given by $2\max \{\ell \; (a),\ell \; (\grave {a})\}$ instead of $\ell \; (a) + \ell \; (\grave {a})$. This is due to the symmetry of path usage when *k*^∗^ is found. We call this lower bound the ***k*-Gravity** of *a*.

It is interesting to note that $\ell \; (a) > \ell \; (\grave {a})$ implies that *ℓ* (*a*) is more frequently involved in shorter paths than $\ell \; (\grave {a})$. For the undirected edge that was replaced by *ℓ* (*a*) and $\ell \; (\grave {a})$, this has implications for higher frequency of the edge’s use in one direction for shorter paths. This also suggests the need for exploration of the use of the algorithm for deeper insights into information transmission and efficiencies, which we leave for future work.

### Illustration of Edge Gravity concepts

We begin by examining simple examples in order to illustrate the details of our methodology and to develop intuition for analysis of the results. For reference, Table 1 provides a list of network examples and case studies to be used in this section and the following.

**Table 1:** Network examples and case studies used in this paper–i.e., a summary of inputs with their sources when applicable

Network example	Nodes (*n*)	Edges (*m*)	Network visualization	Data source
Small example A	5	6	Fig. 1	^*a*^
Small example B	8	10	Fig. 4	^*a*^
Granovetter ’73 example A	10	21	Fig. 7	Granovetter (1973)
Granovetter ’73 example B	25	41	Fig. 8	ibid.
Florentine families	16	20	Fig. 13	Wasserman and Faust (1999)
Florentine families–reduced	16	15	Fig. 13	ibid. ^*b*^
Krebs	19	27	Fig. 18	Krebs (2002)
Krebs–augmented	19	33	Fig. 18	ibid.

The indicator ^*a*^ denotes a network example created for this paper. The indicator ^*b*^ denotes a modification within this paper of the sourced network example

#### Example illustrating path enumeration and the Edge Gravity labels

**Fig. 1:**
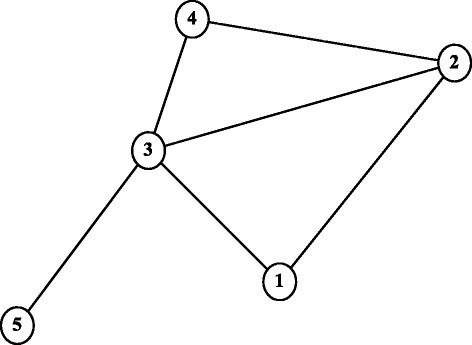
An example used to illustrate the Edge Gravity Algorithm

To illustrate the Edge Gravity Algorithm, the value of the underlying path enumeration, and the related methodology, consider the simple five-node example in Fig. 1. The list of all possible paths in the network–from each node to all other nodes, enumerated while executing the *k*-shortest path solution (see **Main Steps, Step 1**) of the Edge Gravity Algorithm–are shown in Table 2. Note that 58 total paths were found and that *k*^∗^=4. In other words, we found that the Edge Gravity Algorithm terminated with a defined *k*^∗^, and that all 58 paths are enumerated for any evocation of the Edge Gravity Algorithm with *k*≥4.

**Table 2:** Path enumeration for the small example in Fig. 1

From → To	Paths	From → To	Paths	From → To	Paths
1→2	{(1,2)}**	2→4	{(2,4)}**	4→2	{(4,2)}**
1→2	{(1,3),(3,2)}	2→4	{(2,3),(3,4)}	4→2	{(4,3),(3,2)}
1→2	{(1,3),(3,4),(4,2)}	2→4	{(2,1),(1,3),(3,4)}	4→2	{(4,3),(3,1),(1,2)}
1→3	{(1,3)}**	2→5	{(2,3),(3,5)}**	4→3	{(4,3)}**
1→3	{(1,2),(2,3)}	2→5	{(2,1),(1,3),(3,5)}	4→3	{(4,2),(2,3)}
1→3	{(1,2),(2,4),(4,3)}	2→5	{(2,4),(4,3),(3,5)}	4→3	{(4,2),(2,1),(1,3)}
1→4	{(1,2),(2,4)}**	3→1	{(3,1)}**	4→5	{(4,3),(3,5)}**
1→4	{(1,3),(3,4)}**	3→1	{(3,2),(2,1)}	4→5	{(4,2),(2,3),(3,5)}
1→4	{(1,3),(3,2),(2,4)}	3→1	{(3,4),(4,2),(2,1)}	4→5	{(4,2),(2,1),(1,3),(3,5)}
1→4	{(1,2),(2,3),(3,4)}				
1→5	{(1,3),(3,5)}**	3→2	{(3,2)}**	5→1	{(5,3),(3,1)}**
1→5	{(1,2),(2,3),(3,5)}	3→2	{(3,1),(1,2)}	5→1	{(5,3),(3,2),(2,1)}
1→5	{(1,2),(2,4),(4,3),(3,5)}	3→2	{(3,4),(4,2)}	5→1	{(5,3),(3,4),(4,2),(2,1)}
2→1	{(2,1)}**	3→4	{(3,4)}**	5→2	{(5,3),(3,2)}**
2→1	{(2,3),(3,1)}	3→4	{(3,2),(2,4)}	5→2	{(5,3),(3,1),(1,2)}
2→1	{(2,4),(4,3),(3,1)}	3→4	{(3,1),(1,2),(2,4)}	5→2	{(5,3),(3,4),(4,2)}
		3→5	{(3,5)}**	5→3	{(5,3)}**
2→3	{(2,3)}**	4→1	{(4,2),(2,1)}**	5→4	{(5,3),(3,4)}**
2→3	{(2,1),(1,3)}	4→1	{(4,3),(3,1)}**	5→4	{(5,3),(3,2),(2,4)}
2→3	{(2,4),(4,3)}	4→1	{(4,3),(3,2),(2,1)}	5→4	{(5,3),(3,1),(1,2),(2,4)}
		4→1	{(4,2),(2,3),(3,1)}		

All 58 paths were found by executing the Edge Gravity Algorithm with any *k*≥4 (*k*^∗^=4). The subset of shortest paths are indicated by **

**Fig. 2:**
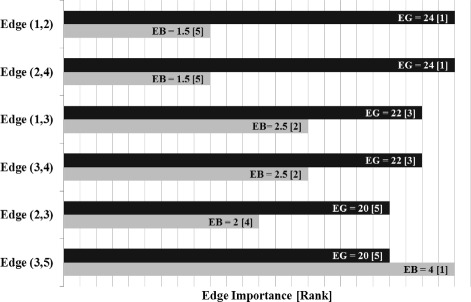
Edge Gravity (EG), Edge Betweenness (EB), and rankings for the network example in Fig. 1

The bar chart in Fig. 2 shows the edge labels and ranking after the Edge Gravity Algorithm terminates, evoked with any *k*≥4. The only bridge in this network is edge (3,5). That is, (3,5) is the only edge whose removal would increase the number of subcomponents; this edge deletion would disconnect node 5 from the rest of the network. However, Fig. 2 shows that according to Edge Gravity, (3,5) is a relatively unimportant edge. This relveals that although edge (3,5) is the most important (and only) relationship for node 5, other edges that rank more highly with respect to Edge Gravity contribute more to the totality of paths in the network and therefore represent relationships that are more structurally important to the potential for information dissemination. For this small example, we designate edge (3,5) as a *bridge to nowhere*.

For this example, edges (1,2) and (2,4) have the greatest Edge Gravity (tied), followed by edges (1,3) and (3,4) (also tied). The most important edges may be formed by these four edges, and removing either sequential pair (1,2) and (1,3) or pair (2,4) and (3,4) would disconnect the network. Note that removing the top two edges, (1,2) and (2,4), would not disconnect the network–illustrating an important distinction between Edge Gravity and cut sets (see Colbourn (1987) for a definition of a network cut set). It is interesting to note that the edges with the highest Edge Gravity are also the edges farthest away from the bridge to node 5.

**Fig. 3:**
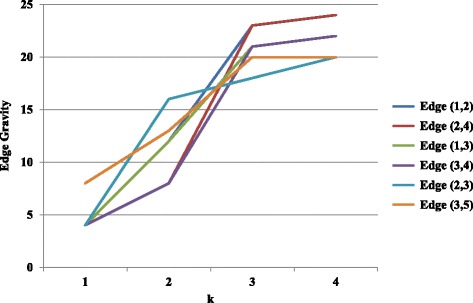
Edge Gravity solutions for the network example in Fig. 1, found by solving successively with *k*=1,2,3,4 (*k*^∗^=4)

To further illustrate the Edge Gravity Algorithm and related methodology, we applied the algorithm successively for *k*=1,2,3,4. Figure 3 shows the progress of the Edge Gravity labels for each edge in this series. In the special case when *k*=1 and all shortest paths are unique, the *k*-shortest path solution is equivalent to the shortest path solution for the network. Shortest paths, used to compute edge betweenness, are indicated by ** in Table 2.

It was easy to enumerate all network paths and thus find *k*^∗^=4. By successively solving the Edge Gravity Algorithm for *k*=1,2,3,4, we were able to illustrate the methodology points regarding the case where *k*^∗^ is not found. First, we observe from Fig. 3 that the Edge Gravity labels are, as expected, monotonically increasing in *k*, which emphasizes their utility as valid lower bound values for Edge Gravity. Next, we observe that the ranking of the edges by gravity score changes for *k*=1,2,3 but is the same for *k*=3 and *k*=4, even though the Edge Gravity magnitudes are different. In this case, solving for *k*=3 would have found the Edge Gravity ranking while not enumerating all network paths.

#### Extending the example: ties that bind and bridges to nowhere

The analysis of the small example in Fig. 1 suggests that a bridge such as (3,5), which connects a network to only one additional node, is not a very interesting bridge. We observed that although the bridging edge is the most important (and only) relationship for node 5, it is the least beneficial to the rest of the network in terms of creating alternate path structure and facilitating information flow. In this sense, we can view edge (3,5) as a *bridge to nowhere*, since the information passing over the bridge has no place farther to travel after reaching node 5. The analysis also raises the issue of how the importance of bridge (3,5) may be impacted by the discovery of additional nodes connected to the bridging node 5. We turn to this question next.

**Fig. 4:**
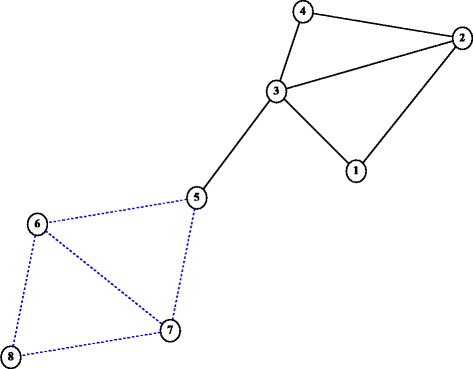
Extension considered for the example of Fig. 1

**Fig. 5:**
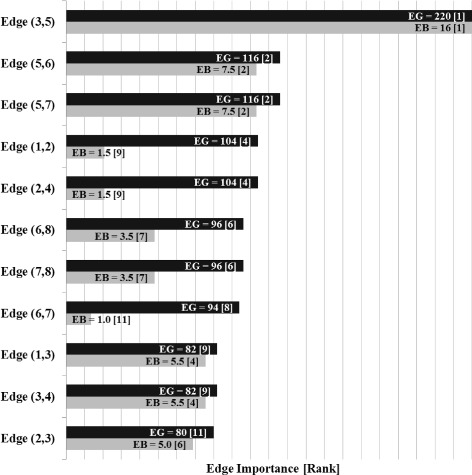
Edge Gravity (EG) and Edge Betweenness (EB) solutions for the example extension in Fig. 4

**Fig. 6:**
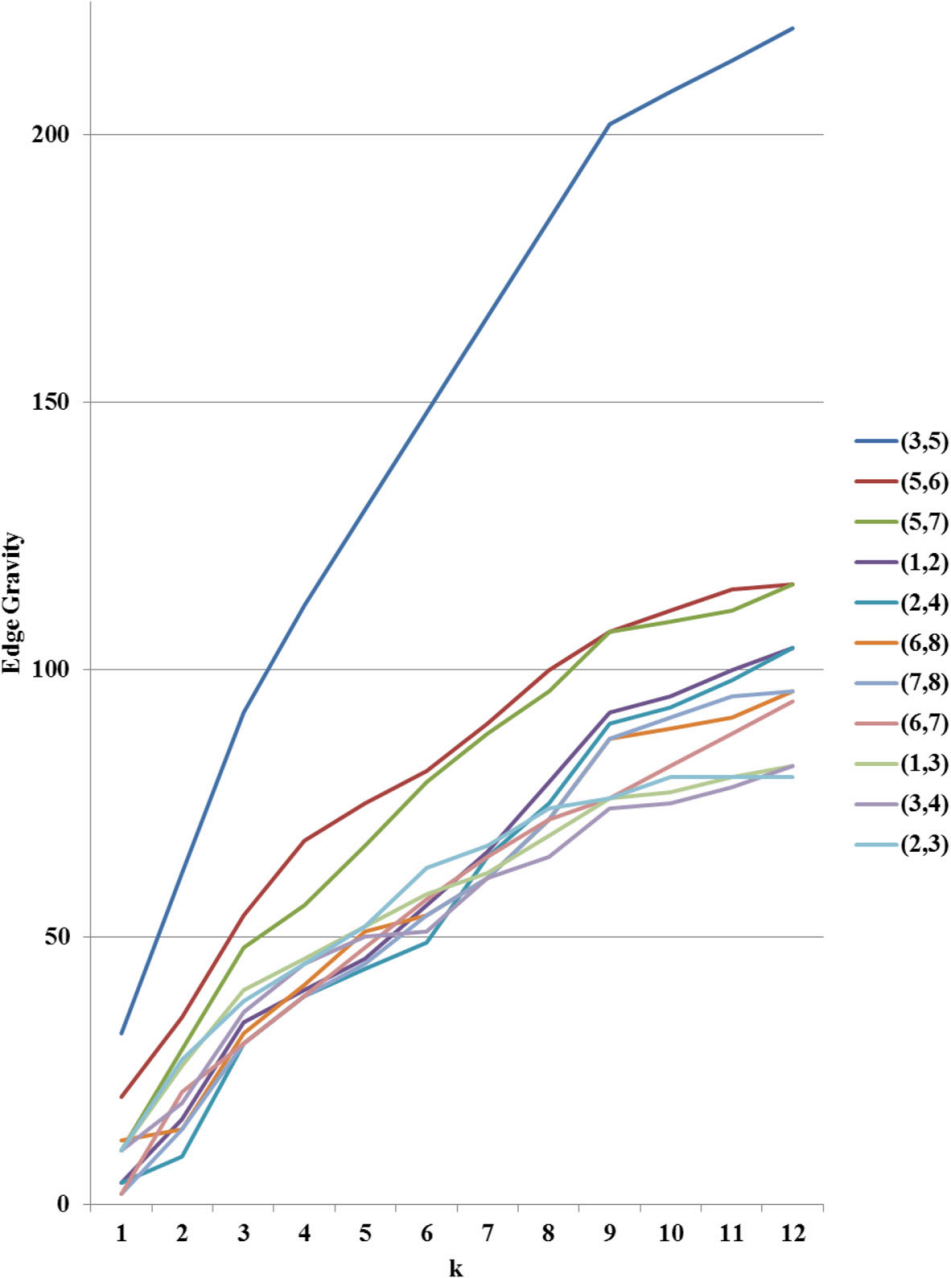
Edge Gravity solutions for the example extension in Fig. 4 when solving the Edge Gravity Algorithm successively for *k*=1,2,3,…,12. *k*^∗^=12

To extend the small example, suppose we discover that the isolated node 5 is indeed connected to additional nodes. We investigate such a scenario in the network represented in Fig. 4. The gravity solutions, as well as the intermediate solutions found by successive iterations of the Edge Gravity Algorithm, converge with *k*^∗^=6 (180 total paths) and *k*^∗^=12 (296 total paths) respectively. In the extended example, we see that edge (3,5) suddenly becomes the most important edge when, as a more interesting bridge, it spans the gap between two groups of nodes that would not otherwise be connected to one another (see Figs. 5 and 6). In the extended example, edge (3,5) may now be considered a *tie that binds*.

## Case studies

Next, we apply the Edge Gravity Algorithm and methodology to several well-known examples from the social network literature. The examples under consideration are the hypothetical examples of Granovetter (1973), the Florentine families network of the Italian Renaissance (Wasserman and Faust 1999), and the Krebs terrorist network (Krebs 2002). We chose to examine these well-known small networks because doing so provides an opportunity to develop insights about the types of structural features that can be revealed by Edge Gravity. Furthermore, the well-known case studies allow us to compare Edge Gravity to previous methodologies, in order to gain a better understanding of the fundamental differences in the way Edge Gravity works compared to existing metrics. In particular, the insights gained allow us to identify and address shortcomings of existing methods, such as edge betweenness.

### Granovetter’s examples

**Fig. 7:**
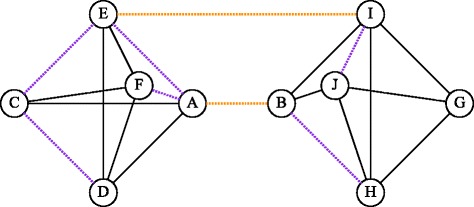
Granovetter’s 1973 Example A network

**Fig. 8:**
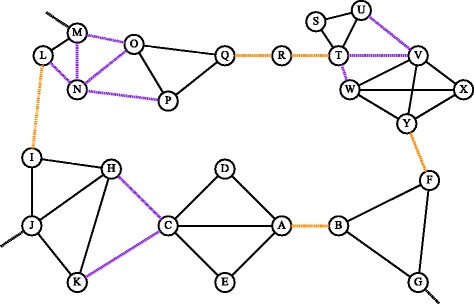
Granovetter’s 1973 Example B network

**Fig. 9:**
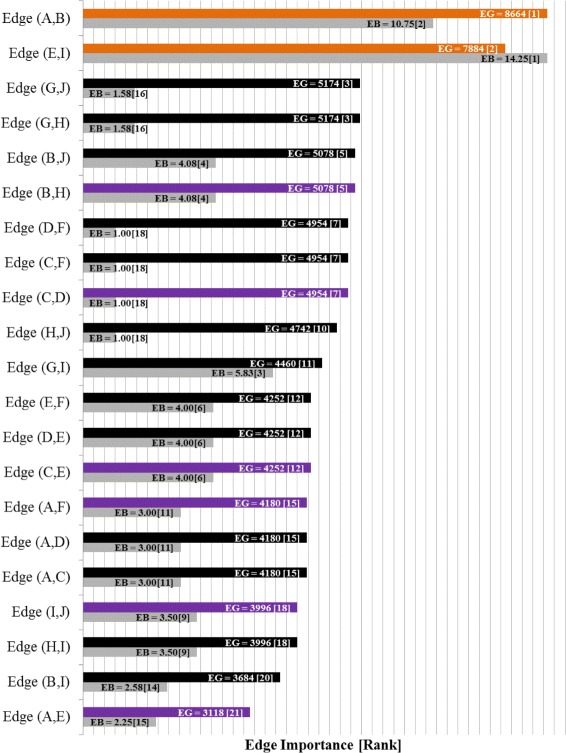
Edge Gravity (EG) and Edge Betweenness (EB) solutions for Granovetter’s Example A

**Fig. 10:**
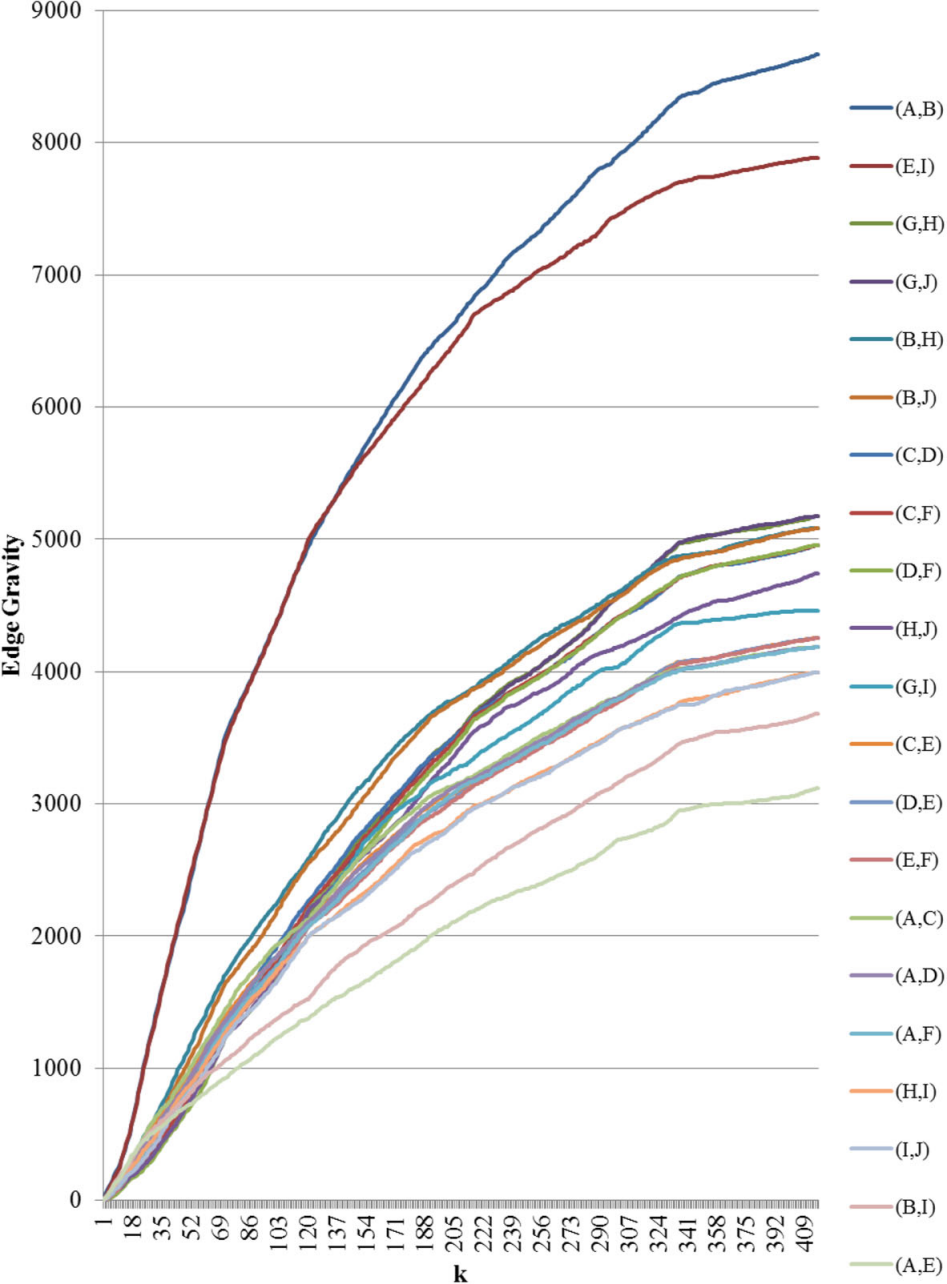
Edge Gravity solutions for Granovetter’s Example A when solving KSP for *k*=1,2,3,…,416

**Fig. 11:**
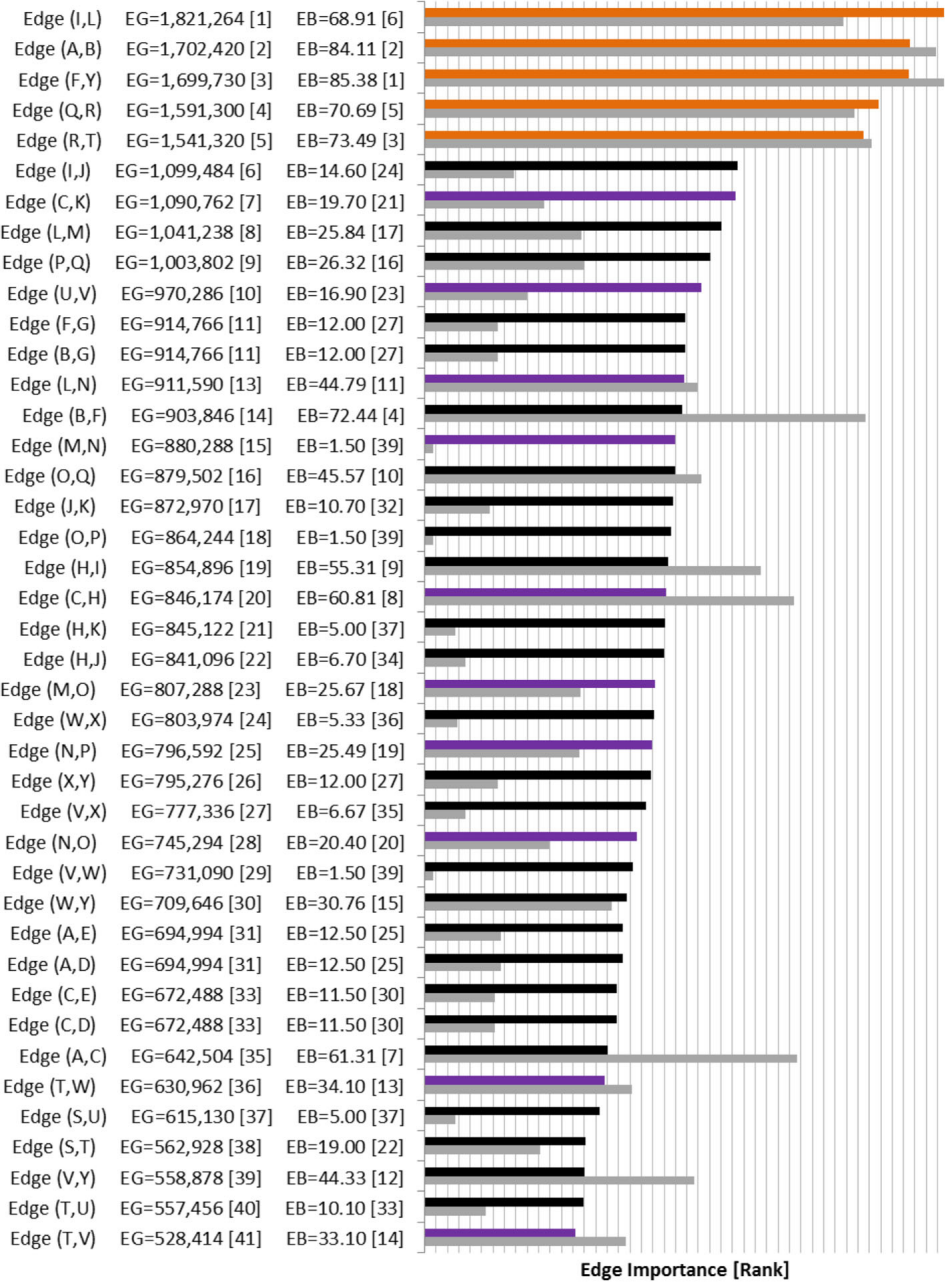
Edge Gravity (EG) and Edge Betweenness (EB) solutions for Granovetter’s Example B

**Fig. 12:**
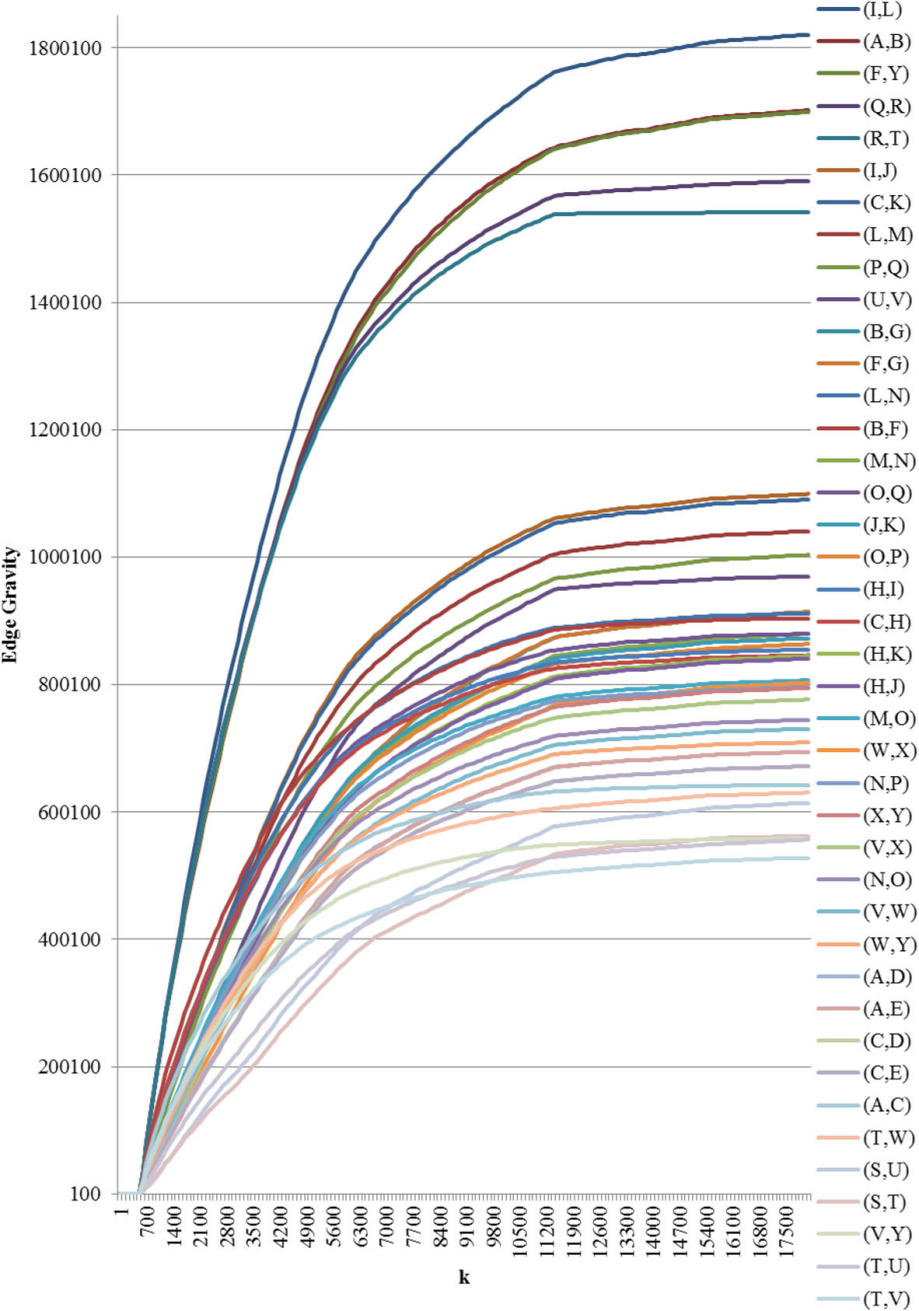
Edge Gravity solutions for Granovetter’s Example B when solving KSP for *k*=1,100,200,300,…,17400,17500

We examine the two network examples given by Granovetter (1973) when he introduced the concept of the strength of weak ties. These examples are given in Figs. 7 and 8. The dashed purple lines represent weak ties that are not local bridges, while the dashed orange represent weak ties that are local bridges. The Edge Gravity labels and ranking for the Granovetter examples can be seen in Figs. 9, 10, 11, and 12.

For Example A (Fig. 7), we see that the edges with the highest Edge Gravity ranking are indeed the local bridges (A,B) and (E,I) discussed by Granovetter. Notice in Figs. 9 and 10 that the local bridges (A,B) and (E,I) are involved in significantly more paths than any other edges in the network. Indeed, edge (A,B) is included in 8664 paths (59% of all paths) and edge (E,I) is included in 7884 paths (53% of all paths). However, the edges ranked third and fourth in this example only appear in 5174 paths each (35% of all paths). This observation supports the validity of Granovetter’s conjecture that local bridges are important because of their involvement in many network paths.

Similarly, for Example B (Fig. 8), we observe that the Edge Gravity Algorithm identifies the local bridges (I,L), (A,B), (F,Y), (Q,R), and (R,T) of Granovetter; the local bridges enjoy the highest ranking and are included in a significantly larger number of total paths than the other edges, as evidenced by Figs. 11 and 12. The local bridges identified by Granovetter in each of these examples are indeed the *ties that bind* the networks together, as demonstrated by the Edge Gravity Algorithm.

Notice that the fourth- and fifth-ranking edges–(Q,R) and (R,T)–are both required to span the gap between node Q and node T. Node R therefore enjoys a high level of brokerage, since any information passing from node Q to node T must pass through node R. Indeed, according to the methods of Valente and Fujimoto (2010) and Everett and Valente (2016), node R ranks highest in terms of brokerage. However, each of these individual edges (Q,R) and (R,T) are ranked lower than the other three local bridges of this example. In other words, the relationships (I,L), (A,B), and (F,Y) are each more structurally important to the network because they are able to reduce distances on their own. Considered individually, the relationships (Q,R) and (R,T) are weaker in terms of their contribution to overall network structure because they must function together, with the help of node R acting as an intermediary broker, in order to transmit information.

Observe that both of Granovetter’s examples include local bridges, but not true bridges. As such, these examples do not contain any *bridges to nowhere*, which can be easily verified by visual inspection of Figs. 9, 10, 11, and 12. Indeed, while the top-ranked *ties that bind* separate themselves significantly from the other edges, no edges stand out at the bottom of the ranking as being included in significantly fewer paths. Instead, the decline in path inclusion among the edges in these examples is gradual, except for the decline following the top-ranked edges (*ties that bind*).

### Florentine families network

**Fig. 13:**
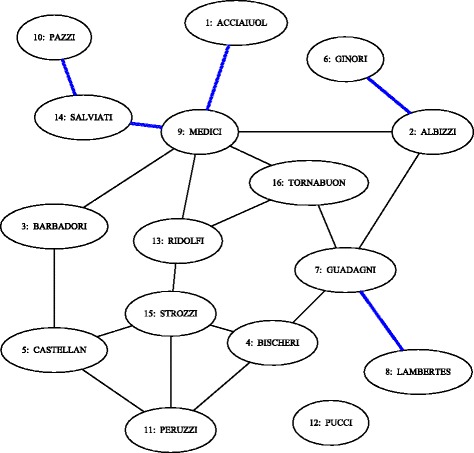
Network model of the Florentine families of the Italian Renaissance

We analyze the classic example of the Florentine families network, following the use of this example by Wasserman and Faust through several chapters of their book (Wasserman and Faust 1999). Illustrated in Fig. 13, the network represents marriage relationships among families with political, economic, and social power during the Italian Renaissance. The Edge Gravity Algorithm was applied, and successfully enumerated all paths (a total of 4128) when executed for any *k*≥33 (i.e., *k*^∗^=33 was found).

**Fig. 14:**
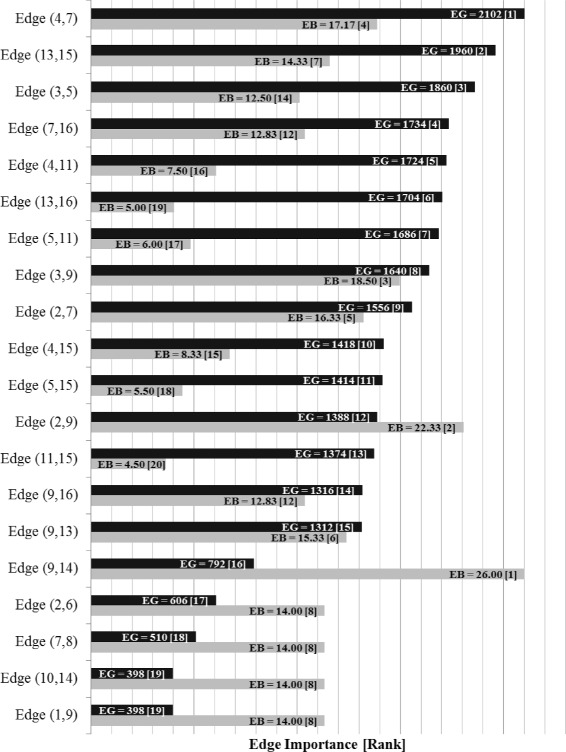
Edge Gravity (EG) and Edge Betweenness (EB) solutions for Florentine families network

Indeed, the bar chart of Fig. 14 shows the final labels and ranking for all edges in the network, as assigned by the Edge Gravity Algorithm. The highest-ranking edge with respect to Edge Gravity–i.e., the edge that appears in the most paths–is (4,7), Bischeri-Guadagni (appearing in 2102 paths, or 51% of all paths). The closest actor to Medici in this pair is Guadagni: The shortest path from Medici to Guadagni has length 2, and the shortest path from Medici to Bischeri has length 3. The second highest-ranking edge with respect to Edge Gravity is (13,15), Ridolfi-Strozzi (appearing in 1960 paths, or 47% of all paths). The edge ranked third is (3,5), Barbadori-Castellan (appearing in 1860, or 45% of all paths). The top three edges, while not individually bridges, can be considered *ties that bind*: Note that if all three are removed, they disconnect two subcomponents of families. Furthermore, in Fig. 13, note that the top three edges are each 4-local bridges.

**Fig. 15:**
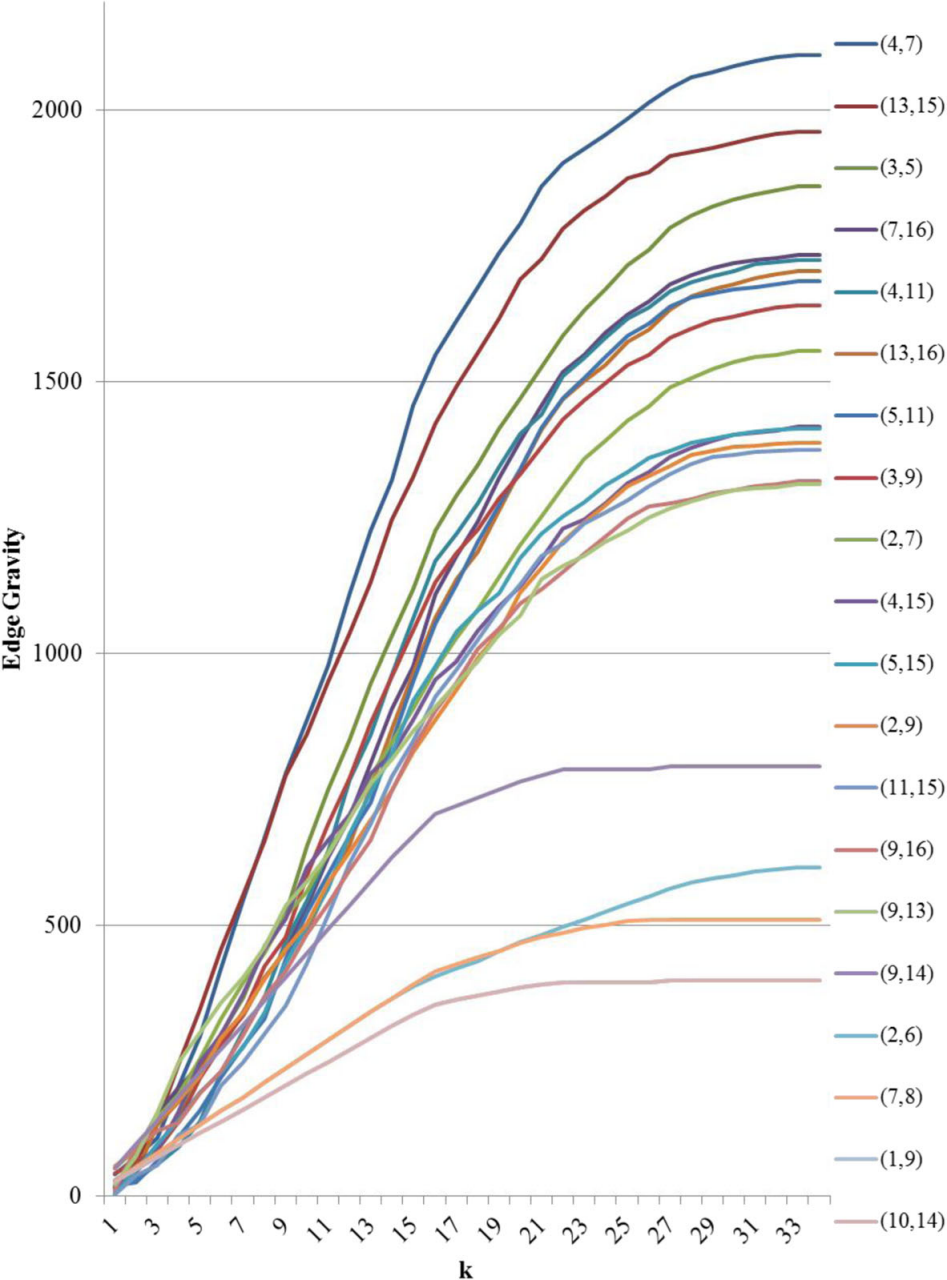
Edge Gravity solutions for the Florentine families network when solving successively for *k*=1,2,3,…,33

Figure 15 reveals that the top three edges (*ties that bind*) as well as the bottom five edges (*bridges to nowhere*) separate themselves in a significant way from the rest of the edges. That is, while “average” edges enjoy a gradual decline of path inclusion, the *ties that bind* stand out as being included in significantly more paths than a typical edge, while the *bridges to nowhere* stand out as being included in significantly fewer.

**Fig. 16:**
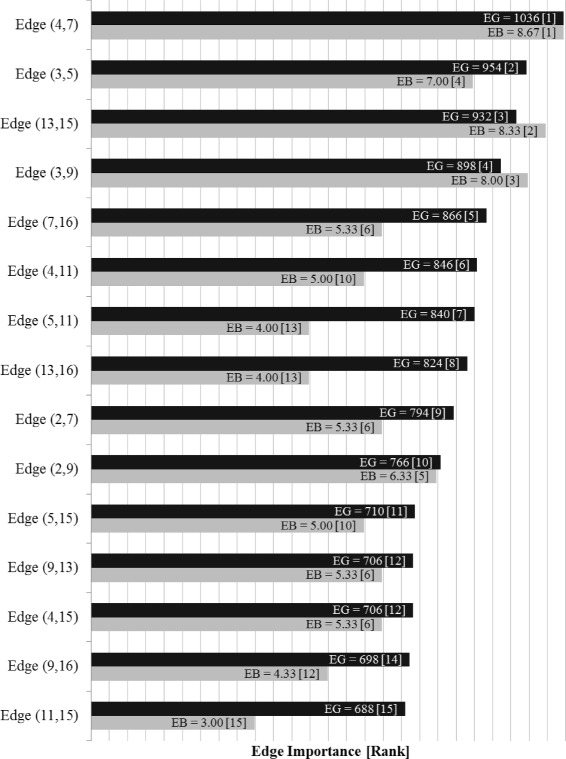
Edge Gravity and Edge Betweenness solutions for the Florentine families network with bridges to nowhere removed

**Fig. 17:**
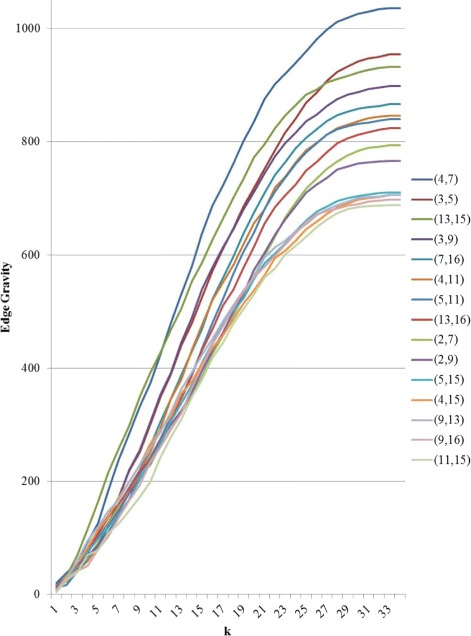
Edge Gravity solutions for the Florentine families network, with bridges to nowhere removed, when solving the Edge Gravity Algorithm for *k*=1,2,3,…,33 (*k*^∗^=33)

The House of Medici, represented by node 9 in Fig. 13, is widely understood to be the most important node in this network. It is well-known that the Medici family ranks highest for several standard social network node centrality analytics. It is interesting to note that according to the Edge Gravity ranking, the top seven edges do not involve the Medici family. Indeed, the Medici family first appears in the edge ranked eighth (out of a total of 20 edges), which is edge (3,9), Barbadori-Medici. Observe that although the Medici family enjoys high node degree and centrality measures, this importance derives in part from edges (10,14) and (1,9), which are *bridges to nowhere*, as well as edge (9,14), which becomes a *bridge to nowhere* after edge (10,14) is removed. These three edges, while important for the individual nodes reached by the bridges, have little impact on the relationship structure of the network as a whole. These edges, as well as edges (2,6) and (7,8), also *bridges to nowhere*, are indicated by blue lines in Fig. 13. We removed the *bridges to nowhere* and reanalyzed the resulting network structure to discover that edge (4,7) remains the highest-ranking edge, while the edges ranked second and third change places with each other. Edge (3,9), Barbadori-Medici, increases slightly in relative Edge Gravity and is ranked fourth in the new network (out of a total of 15 edges). The Edge Gravity Algorithm solution for *k*^∗^=33, found by solving successively for *k*=1,2,3,…,33, is shown in Figs. 16 and 17. Notice that once the *bridges to nowhere* have been removed, no edges stand out as being involved in dramatically fewer paths than the rest.

### Krebs 2001 terrorist network

**Fig. 18:**
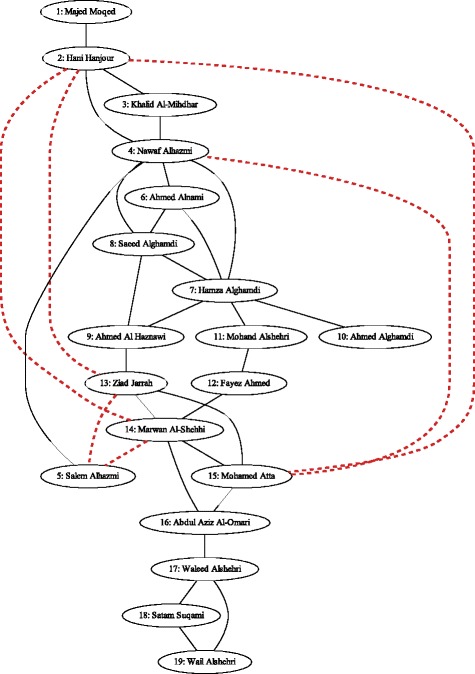
Krebs 2001 terrorist network. To illustrate the augmented terrorist network–the result of additional meetings–red dashed lines were added

Finally, we analyze the terrorist network data set assembled by Krebs (2002), which models the relationships between the 19 hijackers held responsible for the attacks on September 11, 2001. We considered two versions of the network: the original central network as collected by Krebs, and the augmented version in which Krebs introduced another six edges representing contacts that participated in crucial meetings such as the one that took place in Las Vegas. Figure 18 shows both networks, with the original edges in solid black lines and the augmented edges in dashed red lines.

**Fig. 19:**
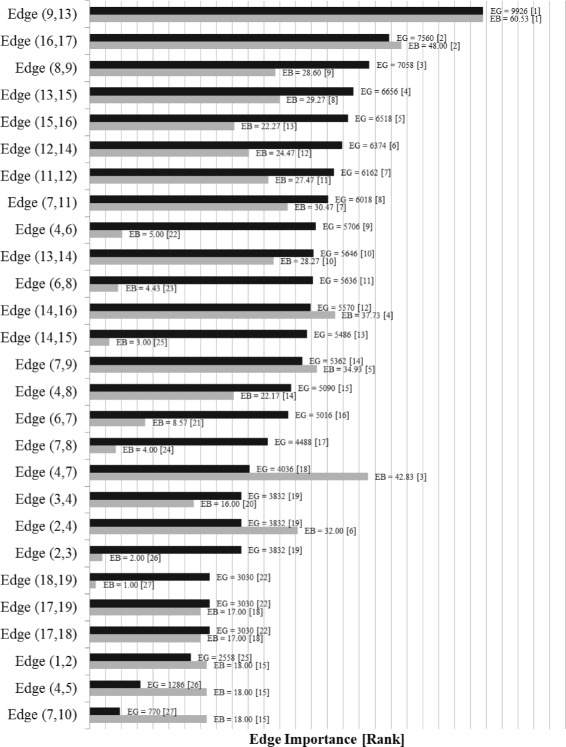
Edge Gravity (EG) and Edge Betweenness (EB) solutions for the original Krebs network

**Fig. 20:**
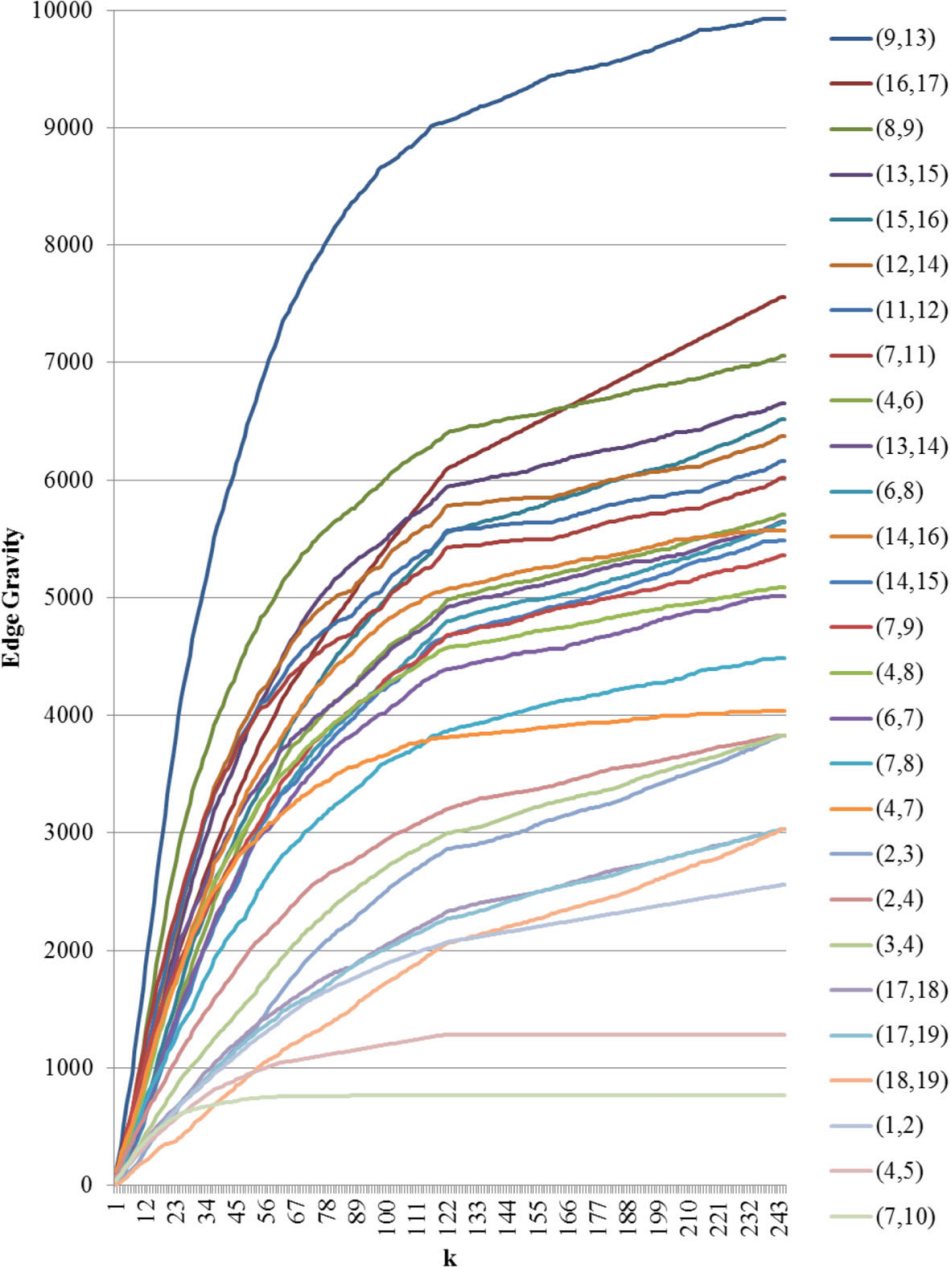
Edge Gravity labels and ranking for the original Krebs network, when solving for *k*=1,2,3,…,244

The Edge Gravity Algorithm labels and ranking for the original Krebs network are given in Figs. 19 and 20. A total of 15,114 paths were found with *k*^∗^=244. Analysis reveals that edge (9,13)–i.e., the edge between Ahmed Al Haznawi and Ziad Jarrah–appears in the most paths. Notice that if the edge between node 9 and node 13 is removed, the next shortest path connecting nodes 9 and 13 has length 5. Thus, edge (9,13) is very important for reducing distances in the network, and is what Granovetter would refer to as a 5-local bridge. The edge appearing in the second-highest number of paths is (16,17)–i.e., the edge between Abdul Aziz Al-Omari and Waleed Alshehri. This edge is a true bridge, whose removal would disconnect the network.

Notice that there is a stark difference in the importance level of these top two edges. Indeed, the highest-ranking edge, (9,13), appears in 65.6% of all paths, whereas the second highest-ranking edge, (16,17), appears in only 50% of all paths. Hence, edge (9,13) is a *tie that binds* and carries a significantly higher importance to the overall path structure than the second-highest-ranking edge, (16,17)–a true bridge whose absence would disconnect the network.

Nodes 4 and 7 rank highly according to several standard node centrality metrics, including degree, closeness, betweenness, reach, and information centrality. However, nodes 4 and 7 are not adjacent to any top-ranking edges according to Edge Gravity. The three lowest-ranking edges in Fig. 19–i.e., edges (4,5), (7,10), and (1,2)–are clearly, from Fig. 18, *bridges to nowhere*. As with the Florentine families case, centrality for nodes 4 and 7 appears to be bolstered by *bridges to nowhere* (4,5) and (7,10).

**Fig. 21:**
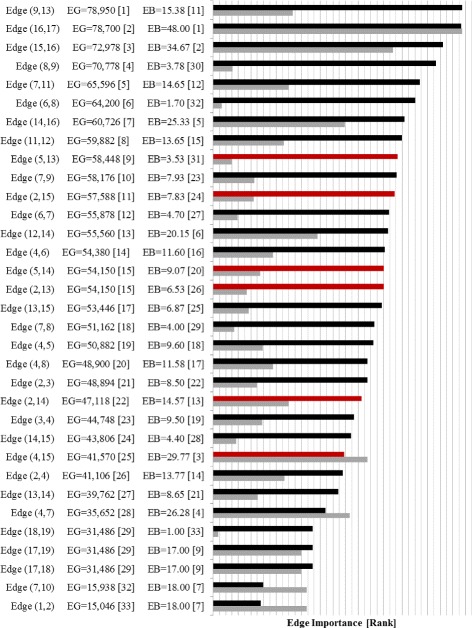
Edge Gravity (EG) and Edge Betweenness (EB) solutions for the augmented Krebs network

**Fig. 22:**
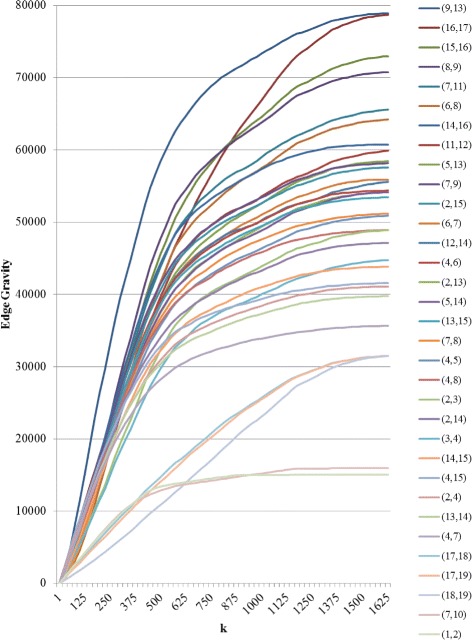
Edge Gravity when solving the k-shortest path (KSP) for the augmented Krebs network, when solving for *k*=1,25,50,75,100,125,150,…,1600,1625

For the augmented version of the Krebs network, a total of 174,588 paths were found and *k*^∗^=1614, as shown in Figs. 21 and 22. The red bars in the bar chart in Fig. 21 represent the added edges for the augmented network. The introduction of the six augmented edges to the network greatly reduces the relative importance of edge (9,13) with respect to Edge Gravity. Nevertheless, edge (9,13) remains the highest ranking, and edge (16,17) remains the second highest ranking. However, while edge (9,13) appears in a total of 78,950 paths (45*%*), edge (16,17) appears in nearly as many: a total of 78,700 paths (45*%*). In the original network, the difference between the number of paths for the top two edges accounts for 15.6*%* of the total network paths; this difference is reduced to only 0.14*%* in the augmented network. Hence, the additional edges in the augmented network changed the path structure significantly enough that both (9,13) and (16,17) serve as *ties that bind*. Inspection of Fig. 18 reveals that the augmented edges create new paths from nodes 17, 18, and 19 to the rest of the graph, many of which bypass edge (9,13). Hence, in the augmented version of the network, the exceptional importance of edge (9,13) relative to edge (16,17) is greatly reduced.

It is interesting to note that the new edges themselves do not carry high Edge Gravity rankings individually, yet their introduction to the network has a significant, measurable impact on the path structure as a whole. Notice that in the series representation in Fig. 20, edge (9,13) separates itself as the clear front-runner early on for small values of *k*. In the augmented version of the network, edge (16,17) ranks highest for edge betweenness based on shortest paths (see Fig. 21); every shortest path from node 17, 18, or 19 to the rest of the graph must include edge (16,17). However, edge (16,17) is middle-ranking for much of the first half of the solution series shown in Fig. 22. The ranking of edge (16,17) increases significantly in the latter portion of the time series. This suggests that many of the new paths including edge (16,17) that were introducted by the augmentation are fairly long.

The augmentation also increased the Edge Gravity ranking of (4,5) to 19th (out of 33 edges), as it is no longer a *bridge to nowhere* after the addition of edges (13,5) and (14,5). Edges (7,10) and (1,2) remain *bridges to nowhere* and continue to exhibit exceptionally low rankings in Figs. 19 and 21. Indeed, observe that the *ties that bind* as well as the *bridges to nowhere* separate themselves in a significant way from the rest of the pack, and do so fairly early in the time series representation.

### Computational results

Table 3 summarizes the computational results for examples and case studies in this paper. The solution was implemented in ANSI standard C, compiled with GCC (https://gcc.gnu.org/), Ubuntu operating system (16.04–64 Minimal for VSI), and executed on an Intel(R) Xeon(R) CPU E3-1270 v6 @ 3.80GHz; 64 GB RAM.

**Table 3:** Summary of computational results, executed on an Intel(R) Xeon(R) CPU E3-1270 v6 @ 3.80GHz

	Edge betweenness		Edge gravity			
Network case	Maximum shortest paths	CPU time (seconds)	*k* ^∗^	Total paths found	Maximum path length	CPU time (seconds)
Small example A	2	0.000060	4	58	4	0.000082
Small example B	2	0.000162	12	296	7	0.000200
Granovetter ’73 example A	4	0.000567	416	14,796	9	0.031664
Granovetter ’73 example B	7	0.006522	17,480	2,130,510	24	124.306396
Florentine families	3	0.000633	33	4128	12	0.004312
Florentine families–reduced	3	0.000269	33	2048	9	0.001798
Krebs	5	0.001920	244	15,114	16	0.031513
Krebs–augmented	5	0.002096	1614	174,588	17	1.152244

## Comparison to other edge metrics

### Edge betweenness

Edge betweenness, first introduced by Girvan and Newman (2002), is an extension of Freeman’s node betweenness to measure edge centrality. Edge betweenness is currently the predominant edge importance metric in social network analysis. The betweenness of an edge (*i*,*j*) is a function of the number of shortest paths that include (*i*,*j*). Multiple shortest paths between a particular pair of vertices are handled in the following way: If there are exactly *m*-many shortest paths between nodes *r* and *s*, then each shortest path is assigned a weight of 1/*m*.

Edge Gravity extends the idea of edge betweenness to include the entire path structure of the network, rather than focusing only on shortest paths. The additional information uncovered by Edge Gravity provides deeper insight into potential communication path structures that may be overlooked by node centrality and edge betweenness metrics based on shortest paths alone. Figures 2, 5, 9, 11, 14, 16, 19, and 21 provide a complete comparison of edge betweenness and Edge Gravity for all of the examples and case studies examined in this paper. Both Edge Gravity and edge betweenness can be computed for disconnected networks–a condition that challenges other metrics, specifically any that rely on the existence of at least one path between every pair of nodes. This is demonstrated by the Florentine families case study (see Figs. 13, 14, and 15, and specifically node 12–the Pucci family).

Analysis of Granovetter’s Example A (Fig. 7) reveals that the local bridges (A,B) and (E,I) of Example A rank highest for both edge betweenness and Edge Gravity metrics, although the order is reversed (see Fig. 9). This illustrates that the structural importance derived from inclusion in shortest paths can be consistent with the structural importance derived from inclusion in all paths quantified by Edge Gravity.

On the other hand, Granovetter’s Example B (Fig. 8) contains five local bridges. Edge Gravity identifies all five local bridges: The top-ranked edges according to Edge Gravity are the local bridges (I,L), (A,B), (F,Y), (Q,R), and (R,T), in that order. On the other hand, edge betweenness fails to include local bridge (I,L) in its top five ranking, even though edge (I,L) appears in more paths than any other edge in the network. Instead, edge (B,F) ranks fourth for edge betweenness yet, by contrast, ranks only 14th for Edge Gravity (see Fig. 11).

A case-by-case analysis of the remaining examples reveals that edge betweenness and Edge Gravity metrics often provide significantly different results from each other. In particular, edge betweenness rankings may be exaggerated by the presence of adjacent *bridges to nowhere*. On the other hand, Edge Gravity ranks the most essential edges without being unduly influenced by the presence of adjacent *bridges to nowhere*.

For the small example described in the “Methodology” section of this paper, edge (3,5) is a *bridge to nowhere* and also ranks highest for edge betweenness (see Figs. 1 and 2). By contrast, the Edge Gravity metric reveals edge (3,5) to be the least important edge to the network path structure as a whole. This is because as a *bridge to nowhere*, it does little to enhance the overall network structure since there is no place farther for information to travel after reaching node 5.

Notice that edge (3,5) appears in every path from node 5 to any other node in the network, since it forms a bridge to the isolated node 5. Therefore, edge (3,5) appears in many shortest paths. However, if we shift our attention to include all paths (Edge Gravity) and not just shortest paths, then the abundance of potential information pathways between nodes 1, 2, 3, and 4 is revealed and the relative structural importance of edge (3,5) is greatly diminished. The low ranking of edge (3,5) according to Edge Gravity reveals its status as a *bridge to nowhere* rather than as a bridge of high structural importance. This example suggests that edge betweenness alone is not sufficient to distinguish structurally important bridges from *bridges to nowhere*; we observe similar results in the Florentine families and Krebs examples below.

Indeed, for the Florentine families example, we see from Fig. 14 that edges (9,14) and (2,9) are the top two edges according to edge betweenness. However, edge (9,14) ranks only 16th according to Edge Gravity, while edge (2,9) ranks 12th. Note also that the top three edges for Edge Gravity rank only fourth, seventh, and 14th for edge betweenness.

Observe that edge (9,14) is a bridge between the main component of the network and a *bridge to nowhere*. It appears that this *bridge to nowhere* places undue importance on edge (9,14) with respect to edge betweenness. Similarly, the edge betweenness rank of edge (2,9) appears to be bolstered by the existence of several bridges adjacent to nodes 2 and 9: (1,9), (2,6), and (9,14). Once the *bridges to nowhere* are removed, as in the example of the reduced Florentine families network, edge betweenness and Edge Gravity both identify (4,7) as the most structurally important edge. In fact, the two metrics identify the same set of edges in their top four rankings, albeit ordered differently.

For the augmented Krebs network, Fig. 21 shows that edge (16,17) ranks highest for edge betweenness and ranks second for Edge Gravity. On the other hand, edge (4,15) is the third-ranking edge according to edge betweenness but ranks only 25th according to Edge Gravity. Similarly, edge (4,7) ranks fourth for betweenness but ranks only 28th with respect to Edge Gravity. Observe that node 7 is adjacent to a *bridge to nowhere*, the presence of which appears to have a boosting effect on edge betweenness for (4,7).

It is interesting to note for the augmented network that although (9,13) is the highest-ranking edge according to Edge Gravity, edge (9,13) ranks only 11th according to edge betweenness. Recall from the case study in the previous section that (9,13) stood out in the original Krebs network as being included in significantly more paths than any other edge. Since (9,13) also enjoyed the highest ranking for edge betweenness, we know that the dramatic boost in path inclusion for (9,13) in the original version was due in part to its inclusion in many shortest paths. The proportion of total path inclusion for (9,13) was greatly diminished in the augmented network, although (9,13) remained the top-ranking edge according to Edge Gravity. This suggests that the introduction of new edges provided shorter alternatives to formerly shortest path structures that depended on edge (9,13). Indeed, the role of edge (9,13) was as a connector between two serpentine ends of the network. Figure 18 shows that the new edges in the augmented version provide shorter alternate routes from opposite ends of the network that bypass edge (9,13).

### The *k*-local bridge metric

The *k*-local bridge metric of UCINET (Borgatti et al. 2002) provides a binary means of identifying bridges and local bridges but does not provide a means of ranking edges in terms of structural importance. The *k*-local bridge metric cannot distinguish a *bridge to nowhere* from a more critically important edge. Similarly, the *k*-local bridge metric cannot distinguish a critically important local bridge from local bridges of lesser importance. It will also overlook structurally important edges that are not bridges or local bridges.

In the Florentine families example (Fig. 13), there are several true bridges, as well as 4-local bridges and 3-local bridges. There are four 4-local bridges in this example: (3,5), (3,9), (4,7), and (13,15). These edges happen to coincide with the top four ranking edges according to Edge Gravity in the reduced version of the example. In the original version of the example, Edge Gravity identifies the 4-local bridges (4,7), (13,15) and (3,5) as the top three ranking edges, while (3,9) ranks eighth.

The original Krebs example (Fig. 18) contains several 5-local bridges: (9,13), (7,11), (11,12), and (12,14). However, only (9,13) emerges as a top-ranking edge for Edge Gravity; edges (12,14), (11,12), and (7,11) rank sixth, seventh, and eighth respectively.

In the augmented version of Krebs, edge (9,13) becomes a 4-local bridge, while the other three remain 5-local bridges. However, edge (9,13) remains most structurally important with respect to Edge Gravity, while the 5-local bridges rank fifth, eighth, and 13th respectively. Instead of these 5-local bridges, Edge Gravity identifies the 4-local bridge (9,13) and the true bridge (16,17) as being the most essential to network path structure.

It is interesting to note that edge (9,13) and the 5-local bridges (7,11), (11,12), and (12,14) are all members of a 6-cycle whose removal would disconnect two serpentine ends of the original network. The edges introduced in the augmented version provide new alternate shortcuts between extreme ends of the network. However, in order to traverse the middle section, information must pass either through edge (9,13) or through the entire edge sequence (7,11), (11,12), and (12,14). Nodes 9 and 13 are both connected to triads; once information leaves either node 9 or node 13, it has multiple paths available to traverse. However, if information begins traveling along the sequence (7,11), (11,12), and (12,14), it must complete this path of length 3 before reaching a node with multiple connections. While the *k*-local bridge metric cannot distinguish between the individual structural importance of these local bridges, Edge Gravity reveals that local bridge (9,13) is more effective at shortening distances between opposite ends of the network than any of the individual local bridges (7,11), (11,12), and (12,14).

Notice that in both versions of the Krebs example, the third- and fourth-ranking edges according to Edge Gravity are neither bridges nor local bridges. To the contrary, these edges are part of triads adjacent to top-ranking edges (9,13) and (16,17). These results indicate that the binary identification of local bridge status alone is not enough to assess or rank edges according to their importance to overall path structure. Indeed, the *k*-local bridge metric focuses on the effect of an edge’s removal on shortest paths but does not consider the larger network structure including all paths. The Edge Gravity metric, in addition to being aligned with Granovetter’s local bridges/weak ties conjecture, provides different and meaningful results because it exploits *all* network paths instead of just the shortest paths.

## Literature review, discussion and conclusions, and future work

### Literature review

Some attention has been given in the literature to the general problem of edge importance, albeit usually not in the context of social network analysis. One approach to edge importance is to study the impact of edge deletion on some network-level property, such as connectivity or diameter. Work in this area includes Barefoot et al. (1987), Bagga et al. (1992), Bollobás (1968), and Boesch et al. (1981), and is used in the study of network reliability and vulnerability. These types of methods seek the minimum number of edges whose deletion is required to disconnect the network or increase its diameter. This differs from our approach in that we seek edges which are individually important to overall network path structure, yet whose individual removal may not necessarily disconnect the network or increase its diameter.

Edge *vitality* identifies the edge whose removal from the network would have the maximum impact on increasing the geodesic distance between a pair of fixed nodes. Papers on *vitality* include: Nardelli et al. (2001), Malik et al. (1989), Lubore and Scilia (1971), and Ball et al. (1989). Our method measures the impact an edge has on all network paths, rather than on a particular shortest path.

The survey paper (Melançon and Sallaberry 2008) compares several edge importance metrics and seeks to organize them into a taxonomy. The focus, however, is on local metrics that are based on network structure in a neighborhood around a particular edge. Our approach, by contrast, is a global edge metric that takes information about the entire network into account.

Hershberger and Suri (2001) considered edge importance, but not in the context of a social network. In their paper, they examined a network model of the Internet, and considered the importance of an edge with respect to pricing in a computer-implemented auctioning algorithm. Hershberger and Suri assumed that shortest paths are always unique–which is generally not a good assumption for social networks, where alternate paths of the same length are both common and desirable.

Most well-known centrality analytics focus on the importance of actors–i.e., the nodes of the network rather than the edges. The canonical example is Freeman’s betweenness centrality (Freeman 1977; 1978), which measures node importance using the frequency in which a particular node appears in a geodesic. Girvan and Newman extended Freeman’s betweenness centrality to define edge betweenness and used the iterative removal of edges to develop algorithmic methods for detecting community structure in Girvan and Newman (2002), Newman and Girvan (2003), and Newman and Girvan (2004). Edge betweenness relies on shortest paths only, rather than considering the global network structure created by all network paths, and can lead to very different results, as explored in the case studies of this paper.

Bonacich (1987) generalized the concept of node centrality by proposing a parameterized family *c*(*α*,*β*) of node centrality metrics. The parameter *β* can be adjusted according to the context of the application: A zero value for *β* correlates to degree centrality, while *β*>0 correlates to conventional centrality metrics such as closeness or betweenness. Negative values of *β* are appropriate for negative exchange scenarios such as bargaining. Each of these metrics is designed to measure the power of an individual, appropriate to a specific context. Rather than focusing on context-specific individual (node) power/centrality, we sought the most important relationship for global network structure and information flow. We observed in the case studies that the most important edges with respect to path inclusion (Edge Gravity) may not be adjacent to the most central nodes.

Holme and Ghoshal noted (Holme and Ghoshal 2008) that there is a cost associated with maintaining social ties. They assert that in contexts such as diplomacy, an actor will want to simultaneously maximize their power and influence (as measured by closeness centrality, which is strongly correlated with degree) while minimizing the number of social ties necessary to maintain. Identifying which ties are the most important and which may be discarded is an interesting problem–and one which can be addressed using the Edge Gravity Algorithm.

Valente and Fujimoto (2010) introduced a metric for node importance that is based on removing edges from a social network and calculating the impact of each edge removal on network cohesion. Every node is then assigned a brokerage metric, the value of which is determined by averaging the effect that each of its adjacent edges has on network cohesion. Everett and Valente modified this approach using betweenness centrality rather than average shortest path length in Everett and Valente (2016). Their approach differs from ours by focusing on node importance rather than on edge importance, and by determining edge relevance to network cohesion using only shortest paths rather than all paths.

We note that social information (e.g., gossip or disease transmission) does not necessarily follow a geodesic route from one person to another. This emphasizes the significance of counting all possible paths instead of just finding the shortest paths. Most of the metrics described in the literature are based on shortest paths (geodesics). However, Stephenson and Zelen (1989) recognized that in a social network, information does not always travel a geodesic route. They proposed a node centrality measure based on the information contained in *all* paths rather than in just the shortest paths. In contrast to our method, Stephenson and Zelen did not enumerate all paths to quantify and rank important edges. Instead, they defined a special matrix to capture the information contained in all paths and used it to define a metric of node (rather than edge) importance.

Recognizing the need to include all paths rather than just geodesics, Alahakoon et al. (2011) introduced a node centrality metric called *κ*-path centrality. This method defines node centrality based on the number of times a node appears in a self-avoiding random walk of length *κ*. De Meo et al. (2012) extended this idea to define a similar *κ*-path centrality metric for edges. De Meo’s algorithm seeks to rank edges according to their potential for enabling information diffusion; the method depends on random walks and is applied to large networks.

The Main Path Analysis method–introduced by Hummon and Dereian (1989)–identifies and ranks important edges in citation networks. Recognizing several limitations to the original method, Liu and Lu (2012) proposed several variations to enhance the methodology. These methods apply only to directed acyclic graphs.

### Discussion and conclusions

Relationships between people are the essence of social networks. The ties between people are what bind a social network together and enable the effective dissemination of information and ideas without predetermined route planning. The social network literature reveals that important information is often transmitted over pathways between people who are not strongly connected to one another and whose personal contacts have little overlap.

Much research has been done to identify important (well-connected, central) nodes in a network. Work has also been done to identify nodes whose removal (along with their ties) would maximally fragment a network or disrupt network cohesion; see Borgatti (2003; 2006). However, both of these approaches view networks through the lens of placing a higher value on individual nodes (people) than on the edges (relationships) between them. These approaches ignore the fact that a particular node’s importance may be drawn from the significance of one of its incident edges, and that the removal of that particular edge–not necessarily the node itself–is the essential action that disrupts the network.

Indeed, when two actors have a relationship to each other that forms a bridge, disrupting the relationship between the two actors may be seen as the key to disrupting the network. Through this lens, the removal of relationships as a by-product of removing actors can be viewed as the essential act in disrupting a network via node removal. Furthermore, when the focus is on network fragmentation, deleting a particular actor might seem significant due to an increase in subcomponents. In contrast, an edge that serves as a local bridge may be more important to overall network communication efficiency, even though its deletion may not create any new subcomponents.

Granovetter observed that local bridges serve as important information pathways by reducing overall distances, yet their removal does not necessarily fragment the network. Local bridges provide shorter, more effective routes for information to be exchanged between actors in different portions of the network, but they are not the only possible way for information to travel. The Edge Gravity Algorithm quantifies Granovetter’s insight that local bridges earn their significance from their inclusion in many paths. The edge that appears most often in any path in the network can thus be viewed as the most structurally important for information diffusion. Edge Gravity identifies the edges that appear in the most paths, and hence have maximum impact on dissemination in terms of reducing distances and enabling potential information flow along many possible alternate paths. Computing Edge Gravity systematically identifies and ranks the most structurally important edges in a social network, the removal of which maximally disrupts potential information flow without necessarily disconnecting the network.

We demonstrated the Edge Gravity Algorithm on a simple hypothetical network in order to better illustrate the concept in practice and to develop intuition for understanding the results. We applied the algorithm to Granovetter’s hypothetical examples, as well as to well-known empirical examples. The algorithm successfully identified essential network edges, and provided a ranking of their relative structural importance to the network.

We found that the most important relationships are often between nodes or actors that do not carry the highest centrality metrics. That is, the most important relationship ties for overall network structure do not always involve the most important, most well-connected, or most central individual people or nodes. To the contrary, the most essential *ties that bind* a network together may be those between actors of average individual importance, as was revealed in the examples of the Florentine families and the Krebs 2001 terrorist networks.

A comparison of Edge Gravity to edge betweenness revealed that although these two metrics may provide similar results under certain circumstances, they can also yield results that are strikingly different. In particular, edge betweenness rankings are susceptible to distortion by the influence of adjacent *bridges to nowhere*. Edge Gravity addresses this shortcoming of edge betweenness. Indeed, the case studies reveal that Edge Gravity identifies and ranks structurally important edges in a stable way that is not skewed by the presence of *bridges to nowhere* connecting to isolated nodes that do little to improve overall network structure and information flow.

Additionally, Edge Gravity effectively identifies *bridges to nowhere*. In each case study, the *bridges to nowhere* were the lowest-ranking edges according to Edge Gravity. In fact, the Edge Gravity scores for *bridges to nowhere* were consistently significantly lower than the scores of other edges, as seen in Figs. 15 and 20. Moreover, the Granovetter B and Krebs examples reveal that Edge Gravity can distinguish structurally important local bridges (ones that reduce distances in the network on their own) from local bridges of lesser importance (ones that require the help of intermediary actors to span a particular gap).

Following the insights of Granovetter (1973), Davies (1966), and also Stephenson and Zelen (1989), we note that social information does not always follow a prescribed route or travel over shortest paths. Granovetter noted that local bridges earn their significance from their inclusion in many paths. The Edge Gravity metric uses all paths rather than just shortest paths, thus providing an opportunity for deeper analysis of this insight.

### Future work

We believe that numerous areas for future work are suggested by the findings in this paper. As with edge betweenness, Edge Gravity has high computational demands. In fact, since Edge Gravity seeks all paths (or many paths, for the bound version), as opposed to just the shortest paths sought by edge betweenness, both execution and memory requirements are naturally more demanding. That Edge Gravity relies on the implicit path enumeration action of *k*–shortest path solution algorithms suggests that focus on computation and memory efficiency in the lower bounding problem is most worthwhile and is likely to be necessary for exploration and study of large networks.

From our examples and case studies, it appears that the Edge Gravity ranking may sometimes be found before *k*^∗^ is determined. An approach such as *Probably Approximately Correct learning* (PAC) may be useful when an accurate edge importance ranking really matters. PAC was first suggested as a computational theory by Valiant in the mid-1980s (Valiant 2013) and has become known in machine learning methodology as a statistical framework for finding polynomially bounded approximate conclusions when the underlying problem is intractable in either memory or computational time; see Shalev-Shwartz and Ben-David (2014).

In this paper, we used known networks to build the intuition of the Edge Gravity metric as well as to compare and contrast with edge betweenness–intuition building that necessitates examination of smaller networks. Preliminary exploration of large networks suggests that there is a trade-off between execution time and active memory, which is required for saving the path lists. Choice of a specific *k*–shortest path algorithm for enumerating the paths appears to be a factor and presents an interesting set of extension studies. Examination and improvement in computation time for Edge Gravity is necessary to address larger static and single-layer networks, which necessarily prefaces complexity that includes time-evolving and multi-layer networks.

The Edge Gravity metric favors edges that are between communities, and because–by design–it embraces alternate paths of all lengths, the importance metric defined by Edge Gravity appears to be better at disfavoring edges within densely connected subgroups. These observations indicate that Edge Gravity, and the Edge Gravity bounds, may be useful building blocks for identifying community structures, extending the work of Girvan and Newman (2002; 2004).

Another area for future work relates to one of the original hypotheses of this study: that there would be a low number of edges in a network that had a very high quantified importance. We found evidence suggesting this phenomenon, which is analogous to the *power law* for node importance (see, for example, Adamic et al. (2001)). However, based on the size of the networks we examined, the evidence is not conclusive. The study of a power law effect for edge importance is left for future work, as it requires the examination of very large networks. For this paper, we chose to focus on well-known networks from the literature, with the intention of gaining insights to complement established understanding. Indeed, we discovered, not unexpectedly, that the Edge Gravity metric placed a high importance on edges that were not adjacent to central nodes.

Finally, we return to a point from the Introduction to this paper: that motivating our research was the notion that in a network, the structural implications of *relationships* are important, and may in fact be more important than the stature of individual nodes. Having reflected upon our work as well as the research of others, we feel that more understanding is needed with respect to *interaction patterns* in social networks. By introducing Edge Gravity, we give a concrete and intuitively interpretable metric that quantifies the structural relevance of an edge without regard for how influential its endpoints are. Given the societal tendency to place undue emphasis on an individual’s prominence, we hope that this will seed more and deeper exploration of the significance of relationships and give these social network components the attention they deserve.
